# Salvage definitive or hypofractionated radiotherapy for oligometastatic recurrence after durvalumab consolidation in limited-stage small-cell lung cancer: a two-case report

**DOI:** 10.3389/fonc.2026.1767015

**Published:** 2026-04-14

**Authors:** Jiahui Shan, Jiayi Yu, Dan Yang, Xiao Chang, Leilei Jiang, Liuhua Long, Yue Teng, Xin Dong, Rong Yu, Huiming Yu, Anhui Shi

**Affiliations:** Key Laboratory of Carcinogenesis and Translational Research (Ministry of Education/Beijing), Department of Radiation Oncology, Peking University Cancer Hospital and Institute, Beijing, China

**Keywords:** case report, durvalumab, hypofractionated radiotherapy, immunotherapy failure, limited-stage small cell lung cancer, oligometastasis, WBRT-SIB

## Abstract

The ADRIATIC trial demonstrated that consolidation durvalumab after concurrent chemoradiotherapy (cCRT) improves progression-free survival (PFS) and overall survival (OS) in limited-stage small cell lung cancer (LS-SCLC), establishing a new standard of care. However, a proportion of patients still develop distant relapse, and optimal salvage strategies after immunotherapy remain unclear. While metastasis-directed radiotherapy, including stereotactic radiotherapy and hypofractionated radiotherapy, has shown benefit in selected oligometastatic non–small cell lung cancer (NSCLC), evidence in SCLC is limited. Here, we describe two LS-SCLC patients who developed limited metastatic relapse after cCRT followed by durvalumab consolidation: one with a solitary adrenal metastasis and the other with two brain metastases. Both patients received salvage radiotherapy (adrenal hypofractionated radiotherapy: 54 Gy in 15 fractions to GTV and 45 Gy in 15 fractions to PTV; brain lesions treated with whole-brain radiotherapy with simultaneous integrated boost [WBRT-SIB]: 40 Gy in 10 fractions to metastatic foci and 30 Gy in 10 fractions to the whole brain). Both patients experienced durable disease control exceeding four years without additional systemic therapy during follow-up. These two cases provide hypothesis-generating clinical observations suggesting that, in carefully selected LS-SCLC patients with limited metastatic relapse, curative-intent local radiotherapy may be feasible and warrants prospective evaluation.

## Introduction

Small cell lung cancer (SCLC) is considered the most aggressive histological subtype of lung cancer. It is classified into limited-stage (LS-SCLC) and extensive-stage (ES-SCLC) disease, with LS-SCLC accounting for approximately one-third of all cases (1). For decades, concurrent chemoradiotherapy (CCRT) has been the standard of care for LS-SCLC patients who are ineligible for or decline surgical intervention (2). However, patient prognosis remains poor, with median progression-free survival (PFS) and overall survival (OS) of approximately 15 months and 30 months, respectively. Only about 25% of patients survive beyond five years after diagnosis, highlighting a significant unmet clinical need in this population (3, 4). The ADRIATIC study provided the first level I evidence that consolidation therapy with durvalumab following CCRT significantly improves both PFS and OS in LS-SCLC. Specifically, durvalumab extended median PFS by 7.4 months and OS by 22.5 months, without increasing treatment-related toxicity (5, 6). This landmark trial has ushered in a new era of immunotherapy for LS-SCLC. Nevertheless, despite these advances, 28% of patients experienced intrathoracic progression and 18% developed distant metastases, emphasizing the persistent need for effective salvage strategies following immunotherapy failure (5).

A key therapeutic challenge lies in managing oligometastatic disease (OMD), a concept first proposed by Hellman and Weichselbaum in 1995, which describes an intermediate clinical state between localized and widely metastatic disease (7, 8). Recent reviews and case-based reports have further explored the clinical implications and management strategies of oligometastatic lung cancer (9–11). In esophageal cancer, OMD is precisely defined—for squamous cell carcinoma, as ≤5 metastases involving ≤3 organs (with no more than 3 in any single organ); and for adenocarcinoma, as ≤3 metastases confined to a single organ or a solitary extranodal site, all of which must be amenable to local treatment (12). In April 2023, the American Society for Radiation Oncology (ASTRO) and European Society for Radiotherapy and Oncology (ESTRO) jointly released the first global clinical practice guidelines for oligometastatic NSCLC. These guidelines define OMD as the presence of ≤5 extracranial metastases, as confirmed by 18F-FDG PET-CT and brain MRI, provided that all lesions are technically treatable with curative-intent local therapy (13). In addition, stereotactic radiotherapy has been defined according to recent ESTRO consensus recommendations as a highly precise, image-guided technique requiring rigorous immobilization, motion management, and quality assurance to safely deliver conformal ablative doses within a limited number of fractions, depending on site- and protocol-specific practice (14).

However, the role of curative-intent local therapy in oligometastatic SCLC remains undefined. Current European Society for Medical Oncology (ESMO) guidelines explicitly state that local treatments are not recommended for metastatic SCLC outside clinical trials (15), due to concerns about rapid systemic dissemination and the presumed futility of localized intervention. This therapeutic nihilism persists despite growing evidence that radiotherapy can synergize with immune checkpoint inhibition by modulating the tumor microenvironment and, in rare cases, inducing immune-mediated abscopal effects (16, 17).

In this context, we present two LS-SCLC cases with limited metastatic relapse after cCRT followed by durvalumab consolidation who experienced long-term disease control after salvage radiotherapy. These cases are intended to provide hypothesis-generating observations and to highlight the potential feasibility of metastasis-directed or definitive local radiotherapy in carefully selected patients, warranting further prospective study.

## Case presentation

### Patient 1

A 55-year-old female lifelong non-smoker was diagnosed with limited-stage small cell lung carcinoma (SCLC, cT2N2M0 per AJCC 8th edition) in October 2020 based on chest CT findings of a left hilar mass with mediastinal lymph node metastases (stations 4L, 7, 8L, 10L) and pathological confirmation, while staging evaluations including brain MRI and abdominal CT revealed no distant spread. On physical examination at diagnosis, the patient was in good general condition with an Eastern Cooperative Oncology Group (ECOG) performance status of 1. No palpable supraclavicular or cervical lymphadenopathy was detected. Cardiopulmonary examination was unremarkable except for mildly decreased breath sounds over the left hilar region. Neurological examination showed no focal deficits.

Between November 2020 and December 2020, she underwent two cycles of first-line EC chemotherapy (etoposide 100 mg/m² d1-3 + carboplatin AUC = 5, d1; q21d), complicated by grade 3 leukopenia after Cycle 1 and grade 2 leukopenia and neutrophils after Cycle 2. From January to February 2021, concurrent thoracic radiotherapy was delivered using 6 MV X-rays via Volumetric Modulated Arc Therapy (VMAT). The Gross Tumor Volume (GTV) comprised the primary left lung lesion (GTVp) and metastatic lymph nodes at stations 4L, 7, 8L, and 10L (GTVn), both prescribed 54 Gy in 30 fractions. A four-dimensional CT scan was used for defining the internal target volume, described as IGTV, prescribed 54 Gy in 30 fractions. A corresponding clinical target volume (CTV) was defined by adding a 5 mm margin in all directions to each IGTV. The Planning Target Volume (PTV) was generated through a 5 mm isotropic expansion from CTV, prescribed 45 Gy in 30 fractions. Cycles 3 and 4 of the EP chemotherapy (etoposide 100 mg/m² d1-3 + cisplatin 75 mg/m² d1-2; q21d) were administered concurrently with radiotherapy on January 9, 2021, and January 29, 2021, respectively, with treatment well-tolerated and no significant toxicities observed.

Post-treatment restaging in March 2021 demonstrated partial response, prompting prophylactic cranial irradiation (PCI; 25 Gy in 10 fractions) (18); durvalumab (1500 mg every 28 days) was initiated in March 2021. She completed five cycles of durvalumab maintenance therapy per protocol (1500 mg intravenously every 28 days) without treatment interruptions or dose modifications.

**Figure 1:**
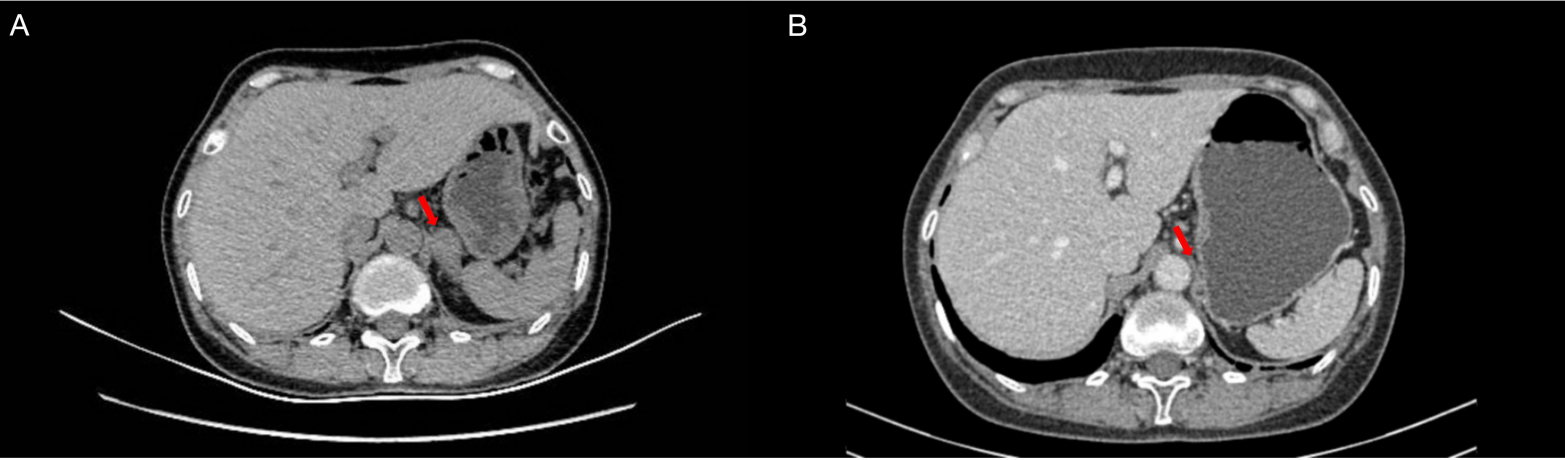
Left adrenal metastasis before radiotherapy **(A)** and after radiotherapy **(B)**.

**Figure 2:**
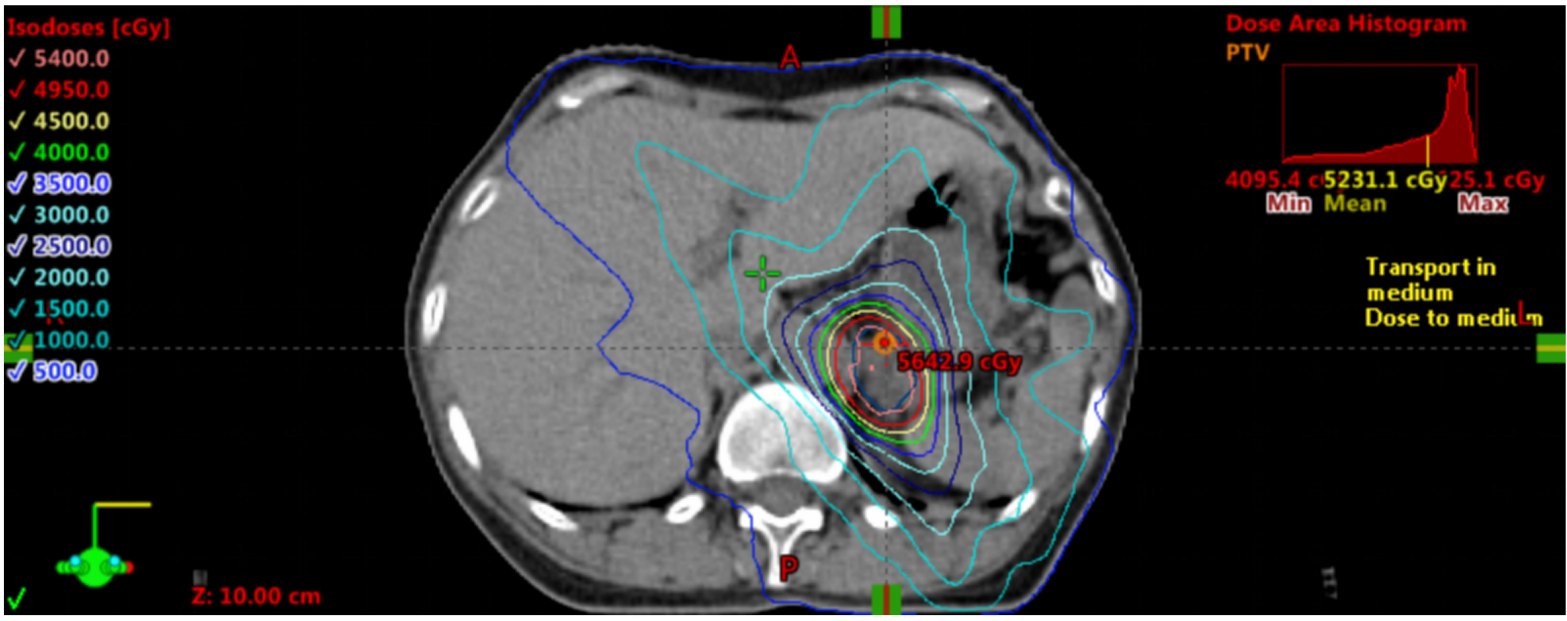
Radiotherapy plan dose distribution displayed on CT (lung window).

Subsequent imaging in August 2021 identified a new 26 × 19 mm left adrenal metastasis (**Figure 1A**) In September 2021, hypofractionated radiotherapy (HFRT) was administered to the adrenal metastasis. The internal gross tumor volume (IGTV), accounting for respiratory motion through 4D-CT delineation, was prescribed 54 Gy in 15 fractions. The gross tumor volume (GTV)—defined as the visible lesion on planning CT—received equivalent coverage (54 Gy/15 fractions), while the planning target volume (PTV, generated by a 5-mm isotropic expansion from IGTV) received 45 Gy/15 fractions (**Figure 2**).

The patient underwent surveillance imaging every 6 months per institutional protocol, with serial chest CT scans demonstrating no evidence of disease progression, while concurrent abdominal CT and brain MRI studies showed no new metastatic lesions (**Figure 1B**). At the 245-week follow-up in June 2025, the patient remained progression-free with excellent functional status (ECOG 0) without additional anticancer therapy, achieving >4-year overall survival from initial diagnosis.

This case illustrates an uncommon clinical course in which durable disease control was observed after salvage hypofractionated radiotherapy to a solitary adrenal metastasis detected after cCRT and durvalumab consolidation. Given the anecdotal nature of a single case and the potential for selection bias, no generalizable conclusions regarding efficacy can be drawn; nevertheless, the prolonged treatment-free interval following local therapy may support further investigation of metastasis-directed radiotherapy in carefully selected patients with limited metastatic relapse.

### Patient 2

A 63-year-old male with limited-stage small cell lung cancer (SCLC, initial stage cT2N3M0 IIIB per AJCC 8th edition) initiated first-line chemotherapy in November 2020 using etoposide (100 mg/m² d1–3 + cisplatin 75 mg/m², d1–2; q21d). At initial presentation, the patient had an Eastern Cooperative Oncology Group (ECOG) performance status of 1 and was in stable general condition. Physical examination revealed no palpable supraclavicular lymphadenopathy. Respiratory examination demonstrated mildly decreased breath sounds over the left lung field without wheezing or rales. No focal neurological deficits were identified at baseline.

**Figure 3:**
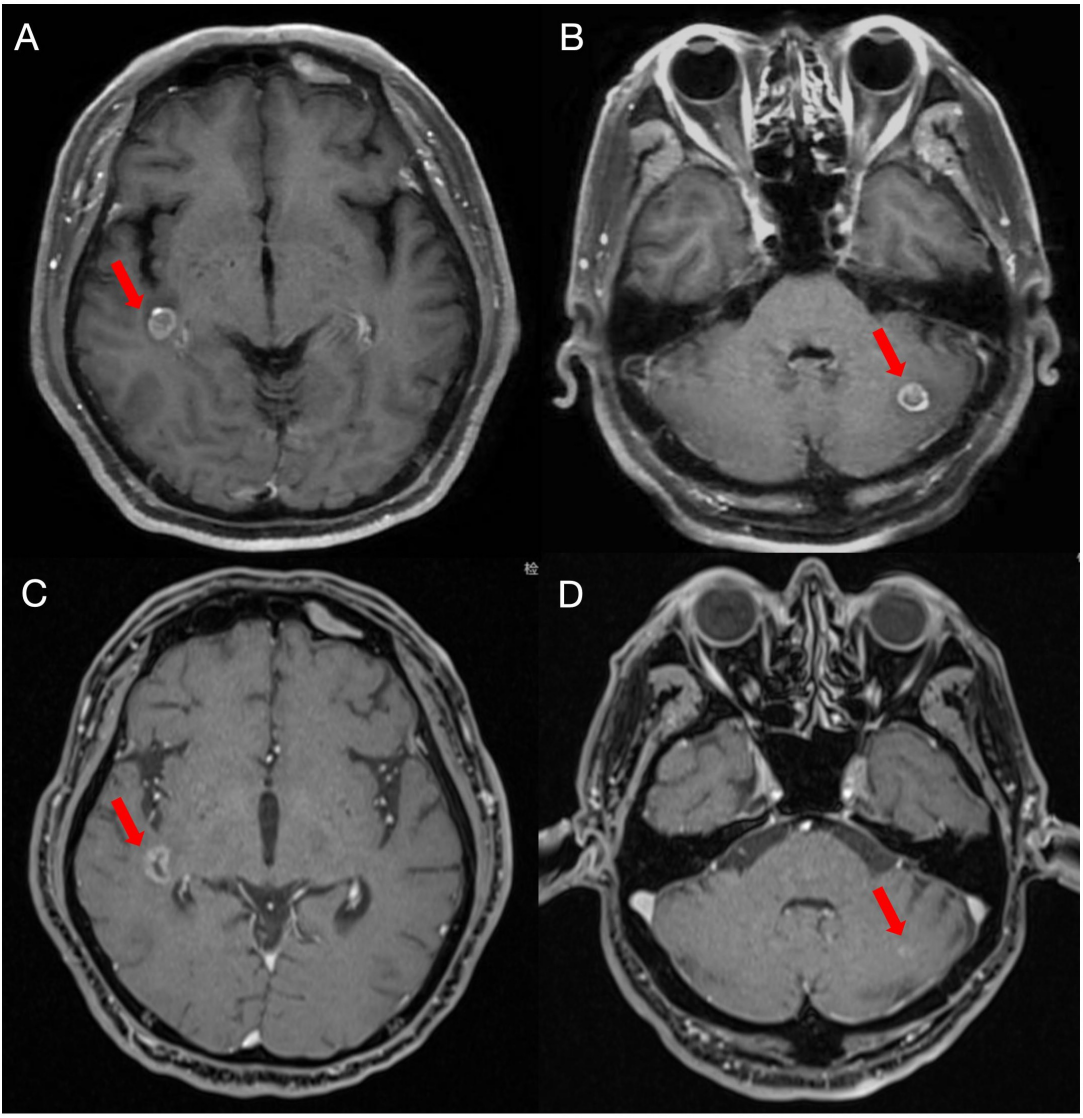
Right temporal lobe metastasis **(A)** and left cerebellum metastasis **(B)** on MRI before radiotherapy. Right temporal lobe metastasis **(C)** and left cerebellum metastasis **(D)** on MRI after radiotherapy.

**Figure 4:**
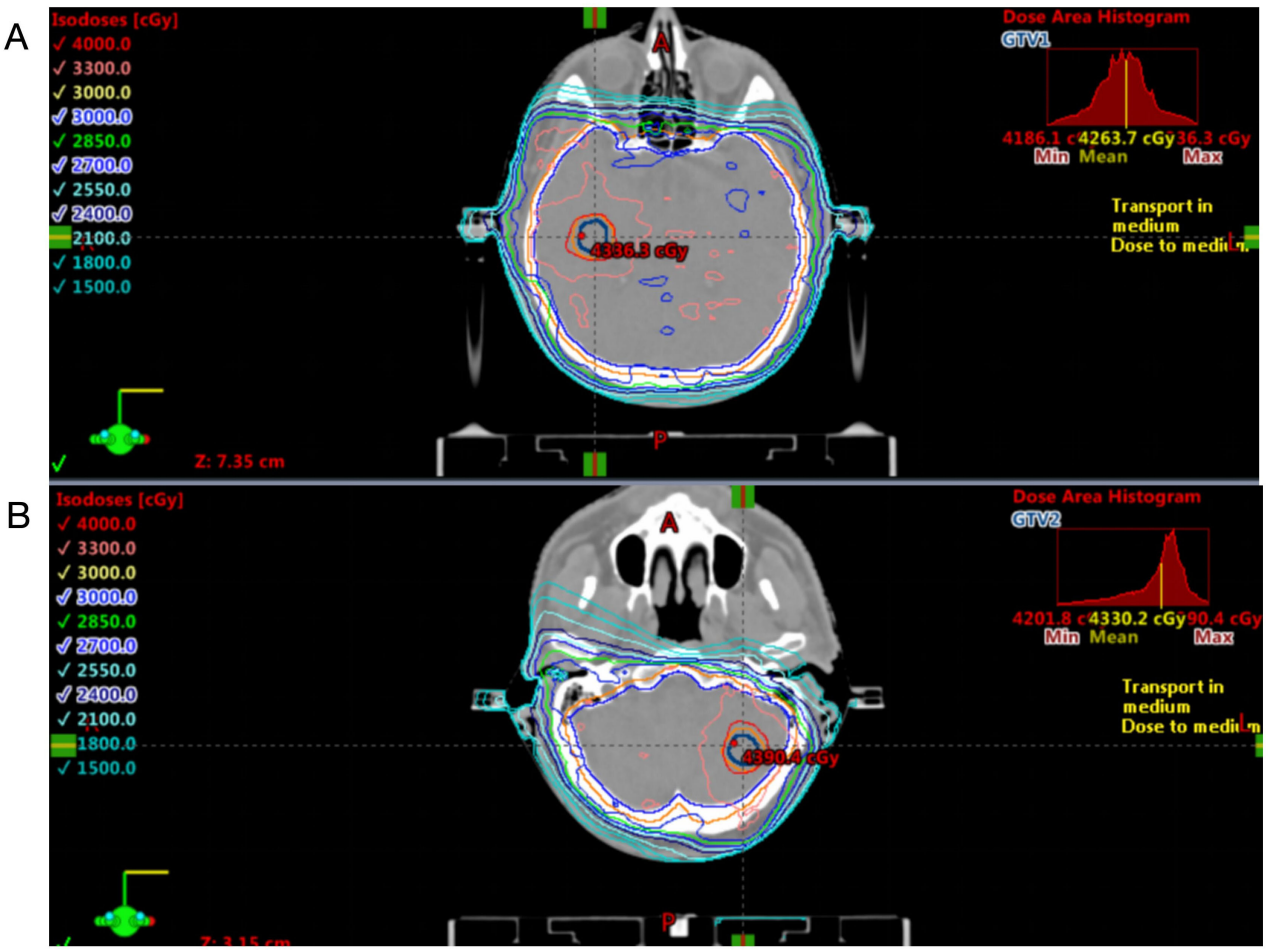
Radiotherapy dose distribution of GTV1 **(A)** and GTV2 **(B)** visualized on planning CT.

Concurrent thoracic radiotherapy was delivered from November to December 2020 using 6 MV X-rays via Volumetric Modulated Arc Therapy (VMAT), with the Gross Tumor Volume (GTV) comprising the primary left lung lesion (GTVp) and metastatic lymph nodes at stations 1L, 2, 4, 5, 6, 7, 8, and 10L (GTVn), both prescribed 54 Gy in 30 fractions. A four-dimensional CT scan was used for defining the internal target volume, described as IGTV, prescribed 54 Gy in 30 fractions. A corresponding clinical target volume (CTV) was defined by adding a 5 mm margin in all directions to IGTV. The Planning Target Volume (PTV) was generated through a 5 mm isotropic expansion from CTV, prescribed 45 Gy in 30 fractions. Cycles 2 of the EP chemotherapy were administered concurrently with radiotherapy. Consolidation chemotherapy cycles 3–4 (December 2020–January 2021) achieved partial remission (PR) but incurred toxicities including grade 2 radiation esophagitis, grade 1 pneumonitis, and chronic bone marrow suppression. The patient declined prophylactic cranial irradiation and received durvalumab (1500 mg Q28d) from February 2021. Subsequent MRI on April 20, 2021, revealed new brain metastases (right temporal lobe and left cerebellum), prompting discontinuation (**Figures 3A, B**). Urgent whole-brain radiotherapy with simultaneous integrated boost (WBRT-SIB) was delivered. Target delineation defined GTV1 as the metastatic lesion in the right temporal lobe and GTV2 as the cerebellar metastasis, with CTV encompassing the entire brain parenchyma (including cerebral hemispheres, cerebellum, and brainstem), expanded isotropically by 3 mm to generate PTV. Treatment was delivered via 6-MV photon volumetric modulated arc therapy (VMAT) with the following dosimetric prescription: 40 Gy in 10 fractions to GTV1 and GTV2, 30 Gy in 10 fractions to CTV, and 30 Gy covering ≥95% of PTV volume (**Figure 4**). Serial follow-up evaluations conducted at 6-month intervals demonstrated sustained absence of disease progression (**Figures 3C, D**). As of June 2025 (245 weeks post-diagnosis), the patient maintained progression-free survival and an overall survival exceeding 56 months—significantly surpassing the median OS of 15–20 months for limited-stage SCLC. This case suggests that WBRT-SIB may achieve durable intracranial disease control in selected patients with limited brain metastases after durvalumab; however, given the inherent limitations of case reports, these observations should be interpreted cautiously and require validation in prospective studies.

## Discussion

This report describes two LS-SCLC patients who developed limited metastatic relapse after standard cCRT followed by durvalumab consolidation—one with a solitary adrenal metastasis and the other with two brain metastases. Both patients received salvage radiotherapy (HFRT and WBRT-SIB, respectively) and experienced durable disease control exceeding four years during follow-up. While these observations are anecdotal and do not allow conclusions regarding efficacy or safety, they may highlight the potential feasibility of curative-intent local therapy in carefully selected LS-SCLC patients with limited metastatic burden (4, 19, 20).

Current guidelines generally discourage routine use of local therapy for metastatic SCLC outside clinical trials, largely due to concerns regarding rapid systemic dissemination. The present cases do not contradict these recommendations but rather underscore the possibility that a small subset of patients may exhibit a more limited metastatic pattern in which local therapy could be considered on an individualized basis, ideally within prospective protocols. In oligometastatic NSCLC, metastasis-directed therapy (MDT), including stereotactic ablative radiotherapy, has been shown to improve progression-free and overall survival (13, 21, 22), and this treatment paradigm has been further discussed in recent clinical reports and reviews of oligometastatic lung cancer (9–11). The 2023 ASTRO/ESTRO guideline established HFRT as a preferred MDT option for appropriately selected oligometastatic NSCLC patients (13). However, similar evidence in SCLC has been lacking. Ongoing prospective efforts are evaluating the integration of radiotherapy in small-cell lung cancer with limited metastatic burden. The RISE phase II randomized trial is investigating radiotherapy integration strategies in extensive-stage SCLC patients with up to 10 metastases (23). Such prospective studies may help clarify the role of metastasis-directed radiotherapy in this biologically heterogeneous disease. Our report suggests that a similar treatment paradigm may apply in selected LS-SCLC cases with true oligometastatic recurrence, warranting prospective validation.

The mechanism underlying these responses may involve immune–radiotherapy synergy, and the sequencing of stereotactic radiotherapy with immune checkpoint inhibitors has been actively discussed in the setting of brain metastases (24). HFRT induces immunogenic cell death, releasing tumor neoantigens and damage-associated molecular patterns (DAMPs), thereby generating an *in situ* vaccine-like effect (25). Radiation also modulates the tumor microenvironment, enhancing T-cell infiltration and reversing immune resistance mechanisms, including those associated with failure of PD-(L)1 blockade (26, 27). The rare but documented “abscopal effect”—regression of unirradiated lesions following localized radiotherapy—supports this immune-mediated response (28).

Although our patients did not receive further immunotherapy after salvage radiotherapy, these findings raise the question of whether reintroducing or continuing immune checkpoint inhibitors (ICIs) after MDT could enhance micrometastatic control or prevent future relapse. Future trials should evaluate this approach, particularly in patients with prior immunotherapy benefit and tolerability (29).

The concept of minimal residual disease (MRD) is also highly relevant. Though standardized MRD biomarkers—such as circulating tumor DNA (ctDNA)—are still under development in SCLC, the durable remission achieved without systemic therapy suggests that MDT may have eradicated all clinically and molecularly evident disease, potentially approximating a state of deep molecular remission (30). Prospective incorporation of MRD monitoring could help identify patients with true oligometastatic biology, confirm complete response post-MDT, and detect early relapse prior to radiologic progression. This would enable a more tailored, biology-driven approach to patient selection and surveillance.

We acknowledge the limitations of case reports, including selection bias and limited generalizability. One patient was a lifelong non-smoker, which may reflect unique tumor biology associated with better prognosis (30). Additionally, both patients had metachronous, limited-site recurrence (1 or 2 metastases), representing a favorable oligometastatic profile. However, the durability of disease control observed after progression during or after durvalumab consolidation suggests that MDT may be considered in carefully selected patients.

In summary, these two cases provide hypothesis-generating observations that durable disease control may be achievable after salvage radiotherapy in carefully selected LS-SCLC patients with limited metastatic relapse following durvalumab consolidation. Prospective clinical trials are needed to define appropriate selection criteria, optimal integration with systemic therapy, and the safety profile of this approach.

## Limitations

This report has several important limitations. First, the findings are based on only two patients and therefore do not allow any generalizable conclusions regarding efficacy or safety. Second, inherent selection bias exists in case reports, and both patients exhibited favorable clinical characteristics, including limited metastatic burden and good performance status, which may not be representative of the broader LS-SCLC population. Third, molecular profiling and minimal residual disease assessments were not available, limiting biological interpretation of the observed outcomes. Finally, although follow-up was prolonged, systematic long-term toxicity assessment remains limited, and the interaction between salvage radiotherapy and prior immunotherapy cannot be fully characterized in this setting. Accordingly, these findings should be interpreted with caution and viewed as exploratory in nature.

## Patient perspective

Both patients reported satisfaction with the treatment outcomes and maintained good quality of life during follow-up. Neither patient reported persistent neurological or systemic symptoms related to salvage radiotherapy. Written informed consent for publication was obtained from both patients.

## Data Availability

The original contributions presented in the study are included in the article/supplementary material. Further inquiries can be directed to the corresponding author.
